# A system review of neoadjuvant immune checkpoint blockade for breast cancer

**DOI:** 10.3389/fimmu.2025.1537926

**Published:** 2025-03-27

**Authors:** Yanle Ye, Zhishan Zhang, Hong Zhao, Bin Zhao

**Affiliations:** ^1^ Central Laboratory, Quanzhou First Hospital Affiliated to Fujian Medical University, Quanzhou, China; ^2^ The Cancer Center of The Fifth Affiliated Hospital of Sun Yat-Sen University, Zhuhai, China

**Keywords:** neoadjuvant therapy, immune checkpoint blockade, breast cancer, toxicity, pathologic complete response, event free survival

## Abstract

**Background:**

The clinical application of immune checkpoint blockade (ICB)-based neoadjuvant therapy has been approved in breast cancer since 2021. However, no studies have evaluated its efficacy and safety in randomized and non-randomized settings. Additionally, there exists controversy about which specific subpopulation can benefit from this management strategy.

**Methods:**

We searched MEDLINE and EMBASE databases for prospective clinical trials of ICB-based neoadjuvant therapy in breast cancer. Information regarding pathological complete response (pCR), event-free survival (EFS), overall survival (OS), and treatment-related adverse event (TRAE) were pooled to estimate the efficacy and safety. Hazard ratio, relative risk (RR) and their 95% confidence intervals (CIs) were calculated.

**Results:**

Among 22 eligible trials including 6134 women with resectable breast cancer, there were 11 randomized studies with 5574 patients. Pooled analysis on pCR (RR, 1.38; 95% CI, 1.20-1.58; *P*<0.001), EFS (hazard ratio, 0.67; 95% CI, 0.54-0.81; *P*<0.001), and OS (hazard ratio, 0.56; 95% CI, 0.35-0.91; *P*=0.01) revealed that ICB-based neoadjuvant therapy was associated with favorable outcomes over conventional treatment. Moreover, the benefits of EFS were independent of PD-L1 expression (*P_interaction_* =0.57) and pCR (*P_interaction_* =0.37) in neoadjuvant immunotherapy. However, combining ICB with conventional neoadjuvant treatment significantly increased the risk of high-grade TRAE (RR, 1.06; 95% CI, 1.01-1.12; *P*=0.03), serious TRAE (RR, 1.57; 95% CI, 1.26-1.94; *P*<0.001), treatment discontinuation due to TRAE (RR, 1.47; 95% CI, 1.14-1.90; *P*=0.003), and potentially fatal adverse event (RR, 2.25; 95% CI, 0.80-6.31; *P*=0.12).

**Conclusion:**

The combination of ICB with conventional neoadjuvant treatment is associated with favorable clinical outcomes and importantly, increased grade 3+ toxicities. Clinicians should meticulously monitor patients to minimize the risk of treatment discontinuation in individuals with potentially curable breast cancer.

## Introduction

Breast cancer (BC) is one commonly diagnosed malignancy and the second leading cause of death among women globally (1). Due to the systematic therapies and surgical advancements, the standard care of paradigm for BC has gradually shifted to neoadjuvant therapy (2). ICB has becomes the standard of care in many cancer types in the past decade (3). In 2021, the United States (US) Food and Drug Administration (FDA) approved the application of ICB-based neoadjuvant therapy for BC due to the improvement in pCR and EFS in KEYNOTE-522 (4). However, it is still unknown which specific subpopulations could benefit from neoadjuvant immunotherapy. Consequently, many studies focus on discovering new biomarkers to spare non-responders from TRAEs. For example, in lung cancer, the European Medicines Agency (EMA) has granted the application of nivolumab-based immunotherapy in the neoadjuvant setting exclusively for patients who exhibit tumor cell PD-L1 expression levels exceeding 1% (5). Interestingly, in BC, the role of PD-L1 expression in neoadjuvant immunotherapy has not been systematically examined.

Key clinical trials have exhibited heterogeneous outcomes for ICB, with some demonstrating significant efficacies resulting in the subsequent approvals, while some revealing safety concerns leading to the early termination. ICB have been associated with a range of morbidities affecting various organs, including cardiovascular, pulmonary, endocrine, neurological, and hepatic systems (6, 7). Additionally, up to 60% of melanoma patients undergoing ICB experience grade 3+ immune-related adverse event (irAE) (8). Occasionally, fatal adverse events (FAEs) are reported (9). Before exclusively relying on ICB, it is imperative to thoroughly understand the clinical implications and address the contentious issues associated with neoadjuvant immunotherapy. A pooled analysis of existing studies can yield critical and clinically relevant insights. Therefore, with recently accumulated evidence, here we systematically evaluate the efficacy and safety of ICB-based neoadjuvant treatment in breast cancer.

## Method

### Search strategy and selection criteria

We searched MEDLINE and EMBASE databases for published trials pertaining to neoadjuvant ICB, alone or in combination, in breast cancer from inception to September 2024 without language restriction. In addition, abstracts from European Society for Medical Oncology conference, American Society of Clinical Oncology conference, and American Association for Cancer Research conference were examined for updates on published trials. The keywords used for search included: breast cancer, clinical trial, immunotherapy, and PD-1/PD-L1. All researchers conducted the search independently, screened the titles and abstracts for relevance, and categorized the potential papers as excluded, included, and uncertain. For uncertain trials, the full-texts were examined to confirm the eligibility.

To be eligible, potential trials had to meet the following criteria: (1) study design: prospective studies irrespective of clinical phase; (2) population: enrolled more than 10 patients, over 18 years of age, had histologic confirmation resectable breast cancer; (3) intervention: at least one arm of patients were treated with neoadjuvant ICB irrespective of dosage or duration; (4) outcomes: pathology (pCR defined as ypT0/is ypN0), efficacy (EFS and OS), and toxicity (TRAE, irAE, serious TRAE, TRAE led to treatment discontinuation, and FAE). Pre-clinical papers, review articles, retrospective studies, editorials, comments, quality of life studies, and cost effectiveness analyses were excluded.

### Data extraction and quality assessment

Relevant data were extracted independently by all investigators using a prespecified form. Extracted data were: (1) study information, including year of publication, first author, clinical phase, study design, definition of endpoints, neoadjuvant regimens, and the sample size; (2) baseline features of the enrolled patients, including age, stage, cancer subtype, and PD-L1 expression; (3) data on treatment-related outcomes, including pCR. The incidence of all-grade and high-grade TRAE, irAE, serious TRAE, and FAE were also recorded. Here, high-grade TRAE meant grade 3+ TRAE. When multiple publications of the same trial appeared, only the most recent or complete report was included.

Risk of bias was assessed by Cochrane risk of bias tool (10) for RCTs and the Joanna Briggs Institute checklist for single-arm trials.

### Statistical analysis

Overall incidences were measured by random-effects or fixed-effects models depending on the heterogeneities. Outcome information from single-arm trials were pooled by an inverse variance random-effects meta-analysis using logit transformation. As for RCT data, we conducted a restricted maximum likelihood meta-analysis of hazard ratios for time-dependent data. Statistical heterogeneities among different studies were estimated by Cochrane’s Q statistic (11). *I* ^2^ statistic was calculated to evaluate the extent of inconsistency contributable to the heterogeneity. The assumption of homogeneity was considered invalid for *P*< 0.05 and *I* ^2^ > 25%.

Publication bias was evaluated through visual inspection of Begg’s funnel plots (12). All analysis was conducted by Stata version 12.0 and MedCalc 18.2.1. Two-sided P<0.05 was considered statistically significant.

## Results

### Baseline characteristics

1097 relevant articles were discovered from the initial search (**Supplementary Figure S1**). After careful review, a total of 22 publications (4, 13–33) and 6 abstracts (34–39) met inclusion criteria, including 22 trials and 6 follow-up studies (4, 24, 29, 36–38), from which we extracted 33 arms. Overall, 6134 patients were included in these 22 trials (median age range, 45-65 years) (**Table 1**, **Supplementary Table S1**). There were 1709 women treated with pembrolizumab, 1091 with atezolizumab, 321 with nivolumab, 218 with durvalumab, 70 with toripalimab, 59 with camrelizumab, and 10 with adebrelimab, and the rest 2656 patients were in control arm. The PD-L1 expression status were recorded in 14 trials (4, 16, 18, 21–25, 28–39), and the detection methods were summarized in **Supplementary Table S2**. Among them, 957 patients with PD-L1 positive tumors and 656 women with PD-L1 negative BC were treated with neoadjuvant ICB, 798 patients with PD-L1 positive tumors and 497 women with PD-L1 negative BC were in the control arms.

**Table 1:** Baseline characteristics of eligible randomized trials.

Study	Masking	Phase	Cancer subtype	Primary endpoint	Treatment	No. of patients	Median age (range, year)	No. of PD-L1+/PD-L1-	median follow-up (months)
GeparNuevo (24, 25)	Double-blind	II	TNBC	pCR	Durvalumab + carboplatin + paclitaxel	88	50 (25-74)	69/9	43.7
					Carboplatin + paclitaxel	86	50 (23-76)	69/11	
IMpassion031 (31, 36)	Double-blind	III	TNBC	pCR	Atezolizumab + nab-paclitaxel	165	51 (22–76)	78/87	39.0
					Nab-paclitaxel	168	51 (26–78)	76/92	
IMpassion050 (32, 37)	Double-blind	III	HER2+	pCR	Atezolizumab + doxorubicin/Cyclophosphamide	228	50	109/119	44.2
					Doxorubicin/Cyclophosphamide	226	50	110/116	43.4
I-SPY2(Pembrolizumab) (26)	Open-label	II	HER2-	pCR	Pembrolizumab + paclitaxel	69	50 (27-71)	NA	33.6
					Paclitaxel	181	47 (24-77)	NA	42.0
I-SPY2(Durvalumab) (27)	Open-label	II	HER2-	pCR	Durvalumab +paclitaxel	73	46 (28–71)	NA	NA
					Paclitaxel	299	48 (24–80)	NA	NA
KEYNOTE-522 (4, 28, 29)	Double-blind	III	TNBC	pCR, EFS	Pembrolizumab + carboplatin + paclitaxel	784	49 (22-80)	656/128	39.1
					Carboplatin + paclitaxel	390	48 (24-79)	317/69	
NCI 10013 (30)	Open-label	II	TNBC	pCR	Atezolizumab + carboplatin + paclitaxel	45	54	16/19	6.6
					Carboplatin + paclitaxel	22	49	4/5	
NeoTRIP (33, 38)	Open-label	III	TNBC	EFS	Atezolizumab + carboplatin + paclitaxel	138	50 (25-79)	79/59	54.0
					Carboplatin + paclitaxel	142	50 (24-77)	77/65	
CheckMate 7FL (34)	Double-blind	III	HR+/HER2-	pCR	Nivolumab + paclitaxel	263	NA	88/169	>12.0
					Paclitaxel	258	NA	84/169	
APTneo (39)	Open-label	III	HER2+	EFS	Atezolizumab + carboplatin + paclitaxel Carboplatin + paclitaxel	448 223	NA NA	201/460	NA
KEYNOTE-756 (35)	Double-blind	III	HR+/HER2-	pCR, EFS	Pembrolizumab +paclitaxel	635	49	482/153	33.2
					Paclitaxel	643	49	489/154	

EFS, event-free survival; pCR, pathological complete response; TNBC, triple-negative breast cancer.

Totally, 11 RCTs were included here, namely GeparNuevo (24, 25), I-SPY2 (Durvalumab) (27), IMpassion031 (31, 36), IMpassion050 (32, 37), KEYNOTE-522 (4, 28, 29), I-SPY2 (Pembrolizumab) (26), NCI 10013 (30), NeoTRIP (33, 38), CheckMate 7FL (34), APTneo (39), and KEYNOTE-756 (35). The enrolled patients on each trial ranged from 67 to 1278. Four trials were phase II RCTs (24–27, 30), and seven were phase III studies (4, 28, 29, 31–39). The primary endpoint was pCR in all RCTs except NeoTRIP (33, 38) and APTneo (39), EFS was co-primary endpoint in KEYNOTE-522 (4, 28, 29) and KEYNOTE-756 (35). The median follow-up on each trial ranged from 6.6 months (30) to 54.0 months (33, 38). Five trials were conducted on 2028 patients with TNBC (4, 24, 25, 28–31, 33, 36, 38), two RCTs on 1799 women with HR+/HER2- BC (34, 35), two studies on 622 patients with HER2- tumors (26, 27), and two trial on 1125 women with HER2+ BC (32, 37). Across 5574 patients included in these RCTs, 2936 patients (52.7%) were treated with ICB, 2638 patients (47.3%) were in the control arms.

Generally speaking, low risks of bias were confirmed in most RCTs (**Supplementary Figure S2**), the major issue was lack of blinding as five studies (26, 27, 30, 33, 38, 39) were open-labelled. For non-RCTs, bias concerns were usually associated with inadequate length of follow-up (**Supplementary Table S3**).

### Efficacy

**Figure 1:**
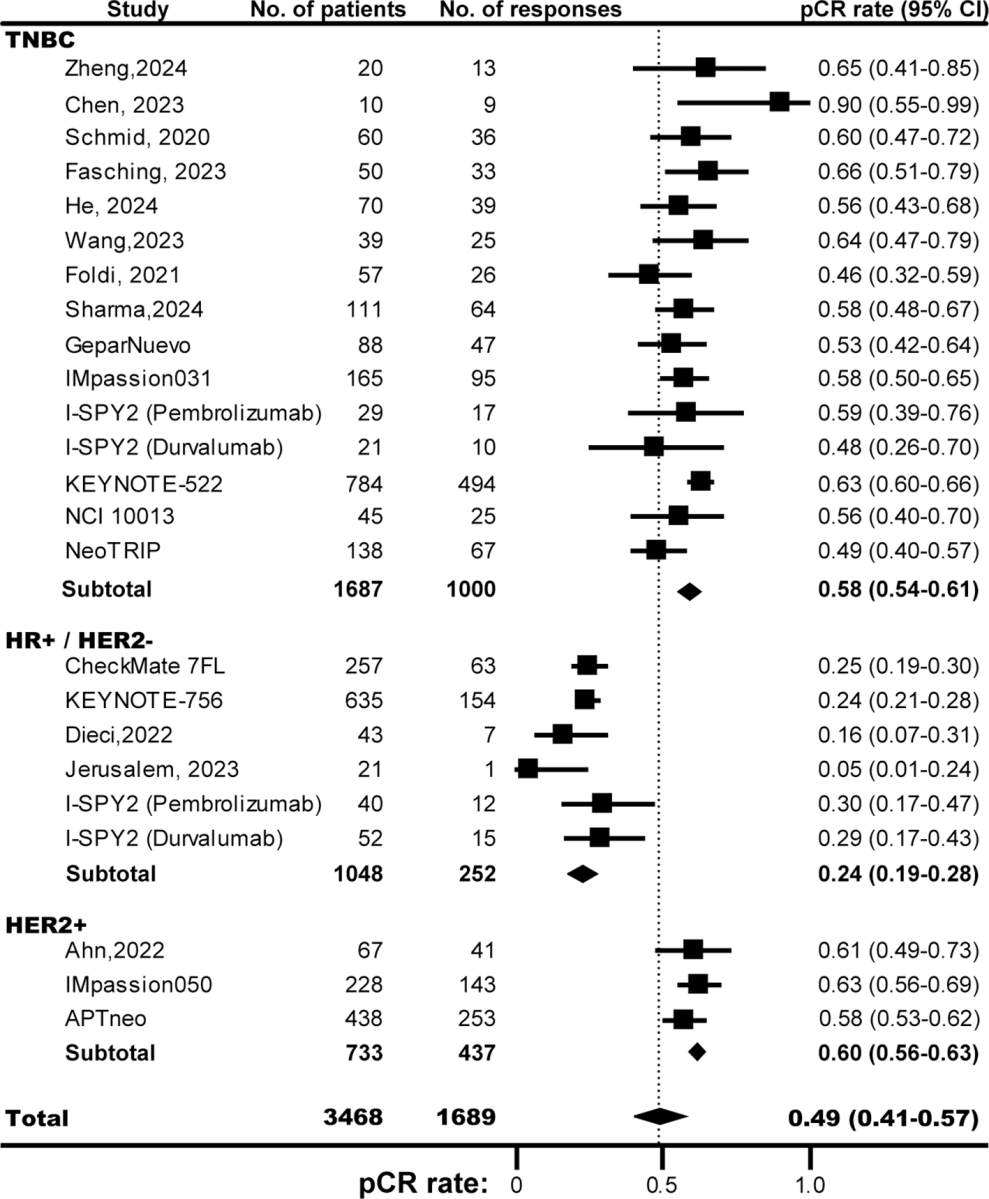
The pooled pCR rates of TNBC, HR+/HER2- BC, HER2- BC, and BC overall in patients treated with ICB-based neoadjuvant regimens. The vertical dot line indicates the overall pCR rate. BC, breast cancer; HER2, human epidermal growth factor receptor 2; HR, hormone receptor; ICB, immune checkpoint inhibitor; pCR, pathological complete response; TNBC, triple negative breast cancer.

For 3468 enrolled patients treated by neoadjuvant immunotherapy, 1689 pCRs were observed (incidence, 49%; 95% CI, 41%-57%; **Figure 1**). Of note, the pCR rates were different among various subtypes of BC, with the highest occurred in HER2+ BC (60%, 95% CI, 56%-63%), and the lowest in HR+/HER2- BC (24%, 95% CI, 19%-28%). The frequency of pCRs in TNBC was 58% (95% CI, 54%-61%). Compared with PD-L1 negative BC (34%, 95% CI, 22%-47%; **Supplementary Figure S3**), the pCR rate in PD-L1 positive tumors (65%, 95% CI, 51%-78%) was almost doubled. For patients treated with anti-PD-1 inhibitors, the pCR rate was 45% (95% CI, 32%-57%), similar with patients treated with anti-PD-L1 inhibitors (54%; 95% CI, 49%-60%; **Supplementary Figure S4**).

**Figure 2:**
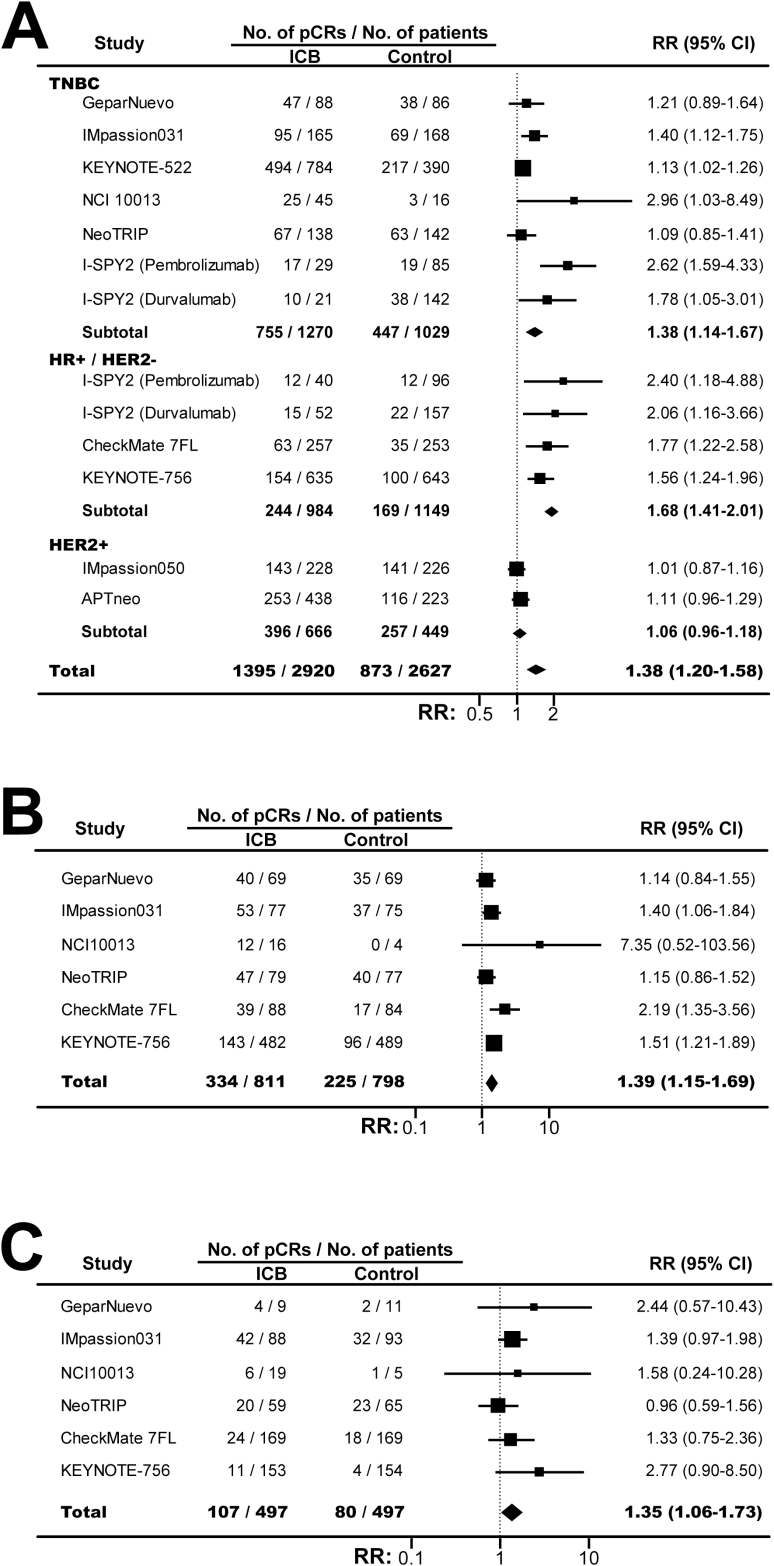
The pooled relative risk of pathological complete response in BC patients treated with ICB-based neoadjuvant regimens. **(A)** The relative risk of pCR in TNBC, HR+/HER2- BC, HER2- BC, and BC overall. **(B)** The relative risk of pCR in patients with PD-L1 positive BC. **(C)** The relative risk of pCR in patients with PD-L1 negative tumors. The vertical dot line equals 1. BC, breast cancer; ICB, immune checkpoint inhibitor; pCR, pathological complete response; RR, relative risk.

Based on 11 RCTs with 5547 patients, ICB-based neoadjuvant therapy was associated with significant increased pCRs (RR, 1.38; 95% CI, 1.20-1.58; *P*<0.001; **Figure 2A**). Interestingly, the increased pCRs were observed in TNBC (RR, 1.38; 95% CI, 1.14-1.67) and HR+/HER2- tumors (RR, 1.68; 95% CI, 1.41-2.01), but not in HER2+ BC (RR, 1.06; 95% CI, 0.96-1.18). Moreover, the pCR rate were significant improved for both PD-L1 positive tumors (RR, 1.39; 95% CI, 1.15-1.69; *P <*0.001; **Figure 2B**) and PD-L1 negative BC (RR, 1.35; 95% CI, 1.05-1.73; *P* =0.01; **Figure 2C**), and the benefits were similar between these two subgroups (*P_interaction_* =0.89).

**Figure 3:**
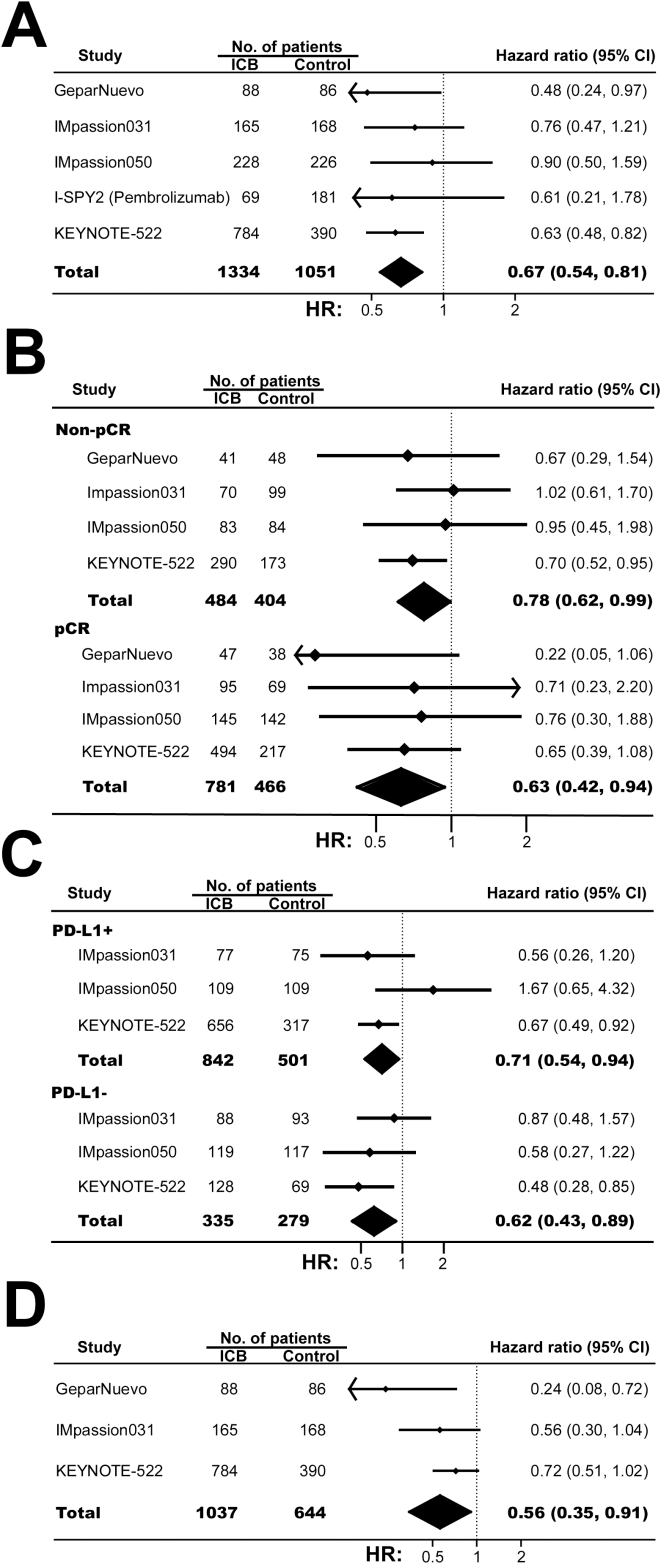
The pooled hazard ratio of survival in BC patients treated with ICB-based neoadjuvant regimens. **(A)** The pooled hazard ratio of EFS in patients treated with ICB-based neoadjuvant regimens. **(B)** The pooled EFS in patients with and without pCR after treated with ICB-based neoadjuvant regimens. **(C)** The pooled hazard ratio of EFS in patients with PD-L1 positive BC and patients with PD-L1 negative tumors. **(D)** The pooled overall survival in patients treated with ICB-based neoadjuvant regimens. The vertical dot line equals 1. BC, breast cancer; EFS, event-free survival; ICB, immune checkpoint inhibitor.

For 2385 patients from 5 RCTs, combining ICB with conventional neoadjuvant therapy was associate with favorable EFS (hazard ratio, 0.67; 95% CI, 0.54-0.81; *P <*0.001; **Figure 3A**). Additionally, similar EFS benefits were observed in patients with pCR and patients without pCR (*P_interaction_* =0.37; **Figure 3B**), or patients with PD-L1+ BC and patients with PD-L1- BC (*P_interaction_* =0.57; **Figure 3C**).

In 1681 women from 3 RCTs, neoadjuvant immunotherapy was associated with favorable overall survival (hazard ratio, 0.56; 95% CI, 0.35-0.91; *P*=0.01; **Figure 3D**). Of note, further investigations were needed to confirm this result since the median follow-ups were 39.1 months, 39.0 months, and 43.7 months for KEYNOTE 522, IMpassion031, and GeparNuevo, respectively.

### Safety

Overall, in BC patients treated by neoadjuvant immunotherapy, the incidence of any-grade TRAE was 99% (95% CI, 98%-100%; **Supplementary Figure S5**); high-grade TRAE, 55% (95% CI, 45%-65%; **Supplementary Figure S6**); any-grade irAE, 34% (95% CI, 24%-45%; **Supplementary Figure S7**); high-grade irAE, 9% (95% CI, 7%-11%; **Supplementary Figure S8**); serious TRAE, 22% (95% CI, 17%-27%; **Supplementary Figure S9**); treatment discontinuation due to TRAEs, 18% (95% CI, 13%-22%; **Supplementary Figure S10**). 12 FAEs were recorded among 3135 patients (incidence, 0.48%; 95% CI, 0.27%-0.78%). The reasons of deaths were septic shock (n=2), myocardial infarction (n=2), alveolitis (n=1), pneumonitis (n=1), hepatitis (n=1), and unknown reasons (n=5).

**Figure 4:**
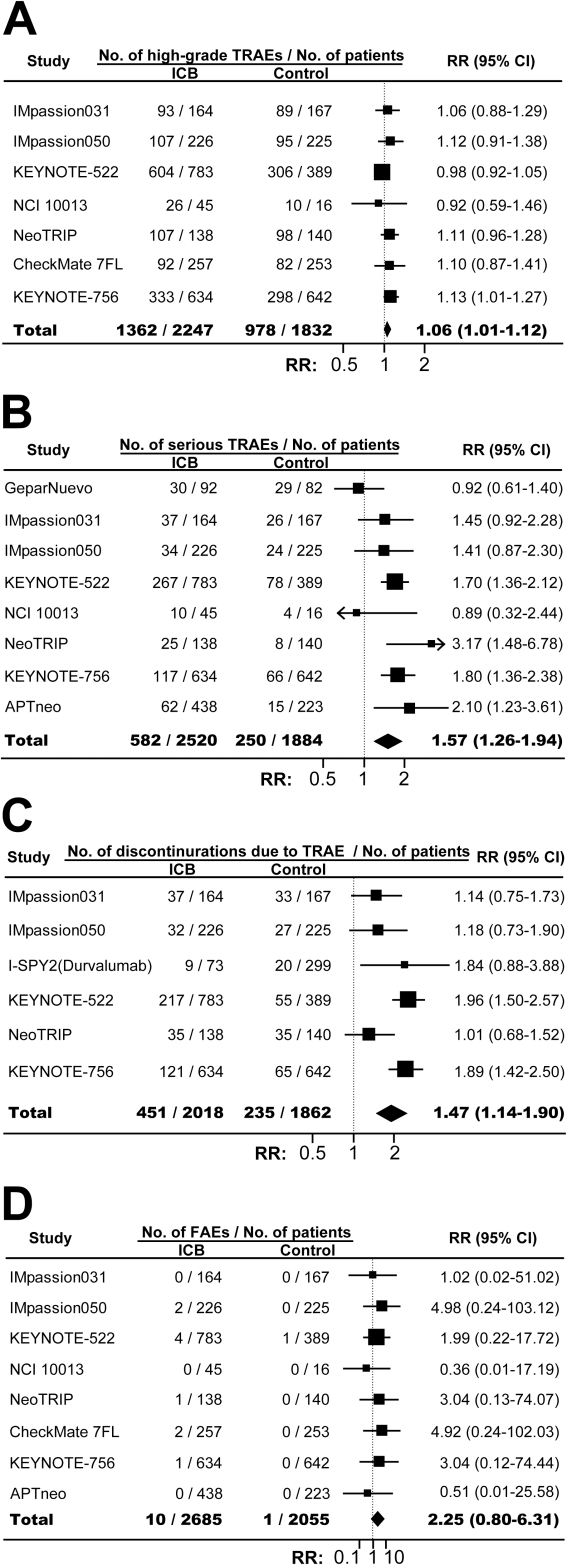
The overall relative risks of toxicities in BC patients treated with ICB-based neoadjuvant regimens. **(A)** The pooled relative risk of high-grade TRAE. **(B)** The pooled relative risk of serious TRAE. **(C)** The pooled relative risk of treatment discontinuration due to TRAE. **(D)** The pooled relative risk of treatment-related fatal adverse events. The vertical dot line equals 1. BC, breast cancer; ICB, immune checkpoint inhibitor; FAE, fatal adverse events; RR, relative risk; TRAE, treatment-related adverse events.

**Figure 5:**
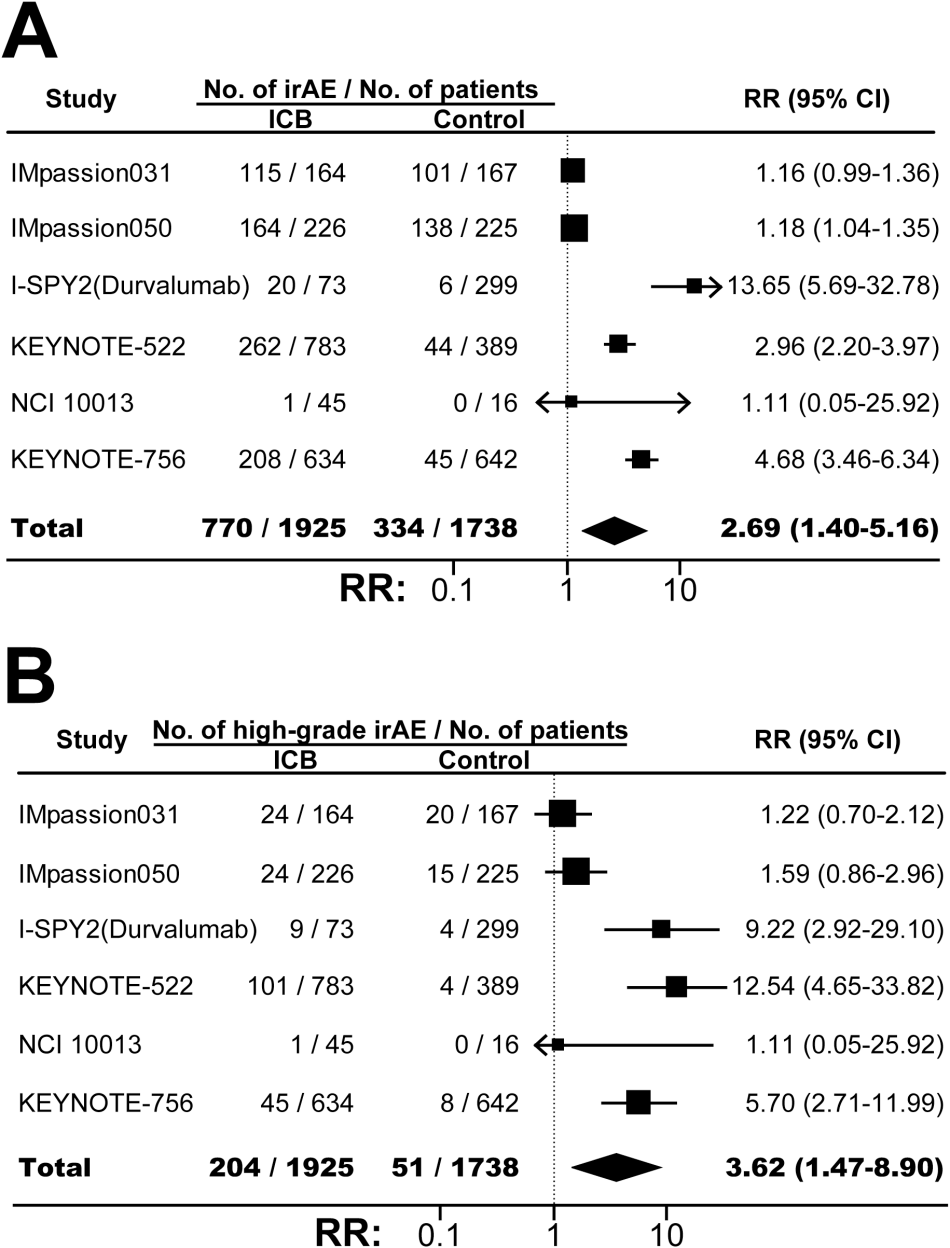
The pooled relative risks of any-grade immune-related adverse events **(A)** and high-grade immune-related adverse events **(B)** in BC patients treated with ICB-based neoadjuvant regimens. The vertical dot line equals 1. ICB, immune checkpoint inhibitor; irAE, immune-related adverse event; RR, relative risk.

The incidences of any-grade TRAE in ICB arm and control arm were similar (RR, 0.99; 95% CI, 0.99-1.00; *P*=0.20; **Supplementary Figure S11**). However, combining ICB with conventional neoadjuvant therapy significantly increased the risk of high-grade TRAE (RR, 1.06; 95% CI, 1.01-1.12; *P*=0.03; **Figure 4A**), serious TRAE (RR, 1.57; 95% CI, 1.26-1.94; *P*<0.001; **Figure 4B**), and treatment discontinuation due to TRAE (RR, 1.47; 95% CI, 1.14-1.90; *P*=0.003; **Figure 4C**). FAE was also increased although the difference was not statistically significant (RR, 2.25; 95% CI, 0.80-6.31; *P*=0.12; **Figure 4D**). As expected, compared with control, ICB-based neoadjuvant therapy was associated with more any-grade irAE (RR, 2.69; 95% CI, 1.40-5.16; *P*=0.003; **Figure 5A**), and high-grade irAE (RR, 3.62; 95% CI, 1.47-8.90; *P*=0.005; **Figure 5B**).

No significant asymmetry was identified by visual inspection of Begg’s funnel plot(**Supplementary Figure S12**).

## Discussion

This study, based on 22 prospective trials involving 6134 patients, estimated the efficacy and safety, and address contentious issues associated with neoadjuvant immunotherapy in breast cancer. Specifically, we examined the pathological complete responses overall and across various molecular phenotypes, investigated long-term outcomes of ICB beyond pCR, evaluated the predictive powers of PD-L1 expression and pCR status as biomarkers, and demonstrated the high risk of toxicities arising from the ICB-based neoadjuvant treatment regimens.

Our study indicated that, compared with conventional neoadjuvant treatment, neoadjuvant immunotherapy could increase the pCR rates in patients with HR+/HER2- and triple-negative breast cancer significantly, but not in HER2+ tumors. Similarly, previous results from KATE2 (40), PANACEA (41), JAVELIN Solid Tumor trial (42), CCTG IND.229 (43), and DS8201-A-U105 (44) also showed poor outcomes of immunotherapy in HER2+ breast cancer with metastatic diseases. Currently, the interactions between anti-tumor immunity and HER2 expression were not fully understood (45). Future studies involving novel immunotherapeutic inhibitors and innovative conjugate antibodies were essential to achieve a more thorough understanding of this specific subtype of BC.

In breast cancer, ICB-based neoadjuvant settings improved the pathological and survival outcomes irrespective of PD-L1 expression status. Similar results had been previously reported in other tumors, such as lung cancer (46). Indeed, it had been well-established PD-L1 expression status was an imperfect biomarker in patient selection and prognostication (7, 47). It appeared more critical to explore the complex interactions between immune cells and cancer cells within the tumor microenvironment, rather than exclusively concentrating on PD-L1 expression. As indicated by the cancer immunogram (48), the efficacy of immunotherapy was influenced by PD-L1 expression as well as a multitude of unrelated characteristics, such as the immune status, the “foreignness” of tumor, the presence of other inhibitory mechanisms, the activity of infiltrated CD8^+^ T cell, and the sensitivity of tumor cells to immune cells. Furthermore, our analysis suggested that among patients undergoing surgery, women that received neoadjuvant ICB had better survival outcomes compared with their counterparts. This finding indicated that the benefits of immunotherapy extended across different stages of disease responses, highlighting its potential in managing breast cancer beyond traditional tumor pathology assessments. On the other hand, since there was no difference in the EFS benefits among BC patients with and without pCR, pCR, might be an imperfect surrogacy for clinical outcomes in neoadjuvant ICB settings.

This study were notable as previous studies failed to reveal the significant association between ICB-based neoadjuvant therapy and toxicities in breast cancer (49, 50). Here we utilized the most up-to-date data to perform this meta-analysis. With more patients included, our study enhanced the statistical power with more robust and reliable outcomes evaluations. Here, our results indicated that high-grade TRAEs occurred in over 50% patients who were treated with the ICB-based neoadjuvant settings. Furthermore, the overall incidence of serious TRAE was 22%, which was doubled the incidence of serious TRAE associated with nivolumab monotherapy (11.2%) (6). The significantly increased grade 3+ TRAEs underscored the necessity for further investigation into patient-specific risk factors that contribute to ICB-related toxicities. Such insights could be pivotal in refining patient selection criteria to mitigate the risk of potentially mortality. Of note, previous studies on patients with advanced-stage solid tumors revealed a potential association between the appearance of TRAEs and favorable survivals (3, 9). However, this concept remained ambiguous in the neoadjuvant context, where toxicities could delay surgery and the consequence clinical outcomes (51).

This study had some limitations. First, while a pooled study with overall survival as the primary outcome should be ideal, it took a prolonged period to obtain sufficient OS information even breast cancer is an aggressive disease. Accordingly, the included trials primarily focus on pCR and EFS rather than OS. Second, the low incidence of treatment-related FAE limited the statistical power to identify any significant differences in this outcome. Third, this study employed a trial-level aggregate meta-analysis of available data, hence the heterogeneity across various trials might not be fully elucidated. The risk factors associated with efficacy and toxicities, the variations in cancer subtypes and antibody structures, dose, and duration of ICB across the included RCTs, were not fully accounted for. Consequently, further examinations should be carried out once individual patient information become available. Of note, safety outcomes were not usually the major target in RCT with a large number of patients, which could diminish the potential publication bias. Fourth, the expressions of PD-L1 in eligible trials were evaluated through several different approaches. Although all these detection methods were approved, the inter-assay discordance was reported (52). This variability could potentially influence the conclusions derived from our analysis.

In summary, for patients with breast cancer, ICB-based neoadjuvant treatment was associated with favorable outcomes, as well as significantly increased grade 3+ toxicities. Additionally, patients with PD-L1 negative BC and patients failed to achieve pCR may also benefit from neoadjuvant immunotherapy.

## Data Availability

The original contributions presented in the study are included in the article/**Supplementary Material**. Further inquiries can be directed to the corresponding author/s.

## Supplementary material

Supplementary Figure 1 Flow-chart diagram of selected clinical trials included in our study.

Supplementary Figure 2 Risk of bias of eligible randomized trials assessed by Cochrane risk of bias tool.

Supplementary Figure 3 The pooled pCR rates in patients with PD-L1 positive BC and patients with PD-L1 negative BC. The vertical dot line indicates the overall pCR rate in patients with PD-L1 positive BC and patients with PD-L1 negative BC. BC, breast cancer; pCR, pathological complete response.

Supplementary Figure 4 The pooled pCR rates in BC patients treated with anti-PD-L1-based and anti-PD-1-based neoadjuvant settings. The vertical dot line indicates the overall pCR rate in patients treated with anti-PD-L1-based and anti-PD-1-based neoadjuvant settings. BC, breast cancer; pCR, pathological complete response.

Supplementary Figure 5 The pooled any-grade TRAE rate in BC patients treated with ICB-based neoadjuvant regimens. The vertical dot line indicates the overall any-grade TRAE rate. BC, breast cancer; ICB, immune checkpoint inhibitor; TRAE, treatment-related adverse event.

Supplementary Figure 6 The pooled high-grade TRAE rate in BC patients treated with ICB-based neoadjuvant regimens. The vertical dot line indicates the overall high-grade TRAE rate. BC, breast cancer; ICB, immune checkpoint inhibitor; TRAE, treatment-related adverse event.

Supplementary Figure 7 The pooled any-grade irAE rate in BC patients treated with ICB-based neoadjuvant regimens. The vertical dot line indicates the overall any-grade irAE rate. BC, breast cancer; ICB, immune checkpoint inhibitor; irAE, immune-related adverse event.

Supplementary Figure 8 The pooled high-grade irAE rate in BC patients treated with ICB-based neoadjuvant regimens. The vertical dot line indicates the overall any-grade irAE rate. BC, breast cancer; ICB, immune checkpoint inhibitor; irAE, immune-related adverse event.

Supplementary Figure 9 The pooled serious TRAE rate in BC patients treated with ICB-based neoadjuvant regimens. The vertical dot line indicates the overall high-grade TRAE rate. BC, breast cancer; ICB, immune checkpoint inhibitor; TRAE, treatment-related adverse event.

Supplementary Figure 10 The pooled incidence of treatment discontinuation due to TRAE in BC patients treated with ICB-based neoadjuvant regimens. The vertical dot line indicates the overall treatment discontinuation rate. BC, breast cancer; ICB, immune checkpoint inhibitor; TRAE, treatment-related adverse event.

Supplementary Figure 11 The pooled relative risk of any-grade TRAE in BC patients treated with ICB-based neoadjuvant regimens. The vertical dot line indicates 1. BC, breast cancer; ICB, immune checkpoint inhibitor; RR, relative risk; TRAE, treatment-related adverse event.

Supplementary Figure 12 Begg’s funnel plot for the publication bias test. Each circle represents a separate trial for the indicated association. Vertical line, mean effect size.
