# Supplementary figures and images for: A system review of neoadjuvant immune checkpoint blockade for breast cancer
Source: Front Immunol. 2025 Mar 27;16:1537926. doi: 10.3389/fimmu.2025.1537926 (PMC11983617; doi:10.3389/fimmu.2025.1537926)

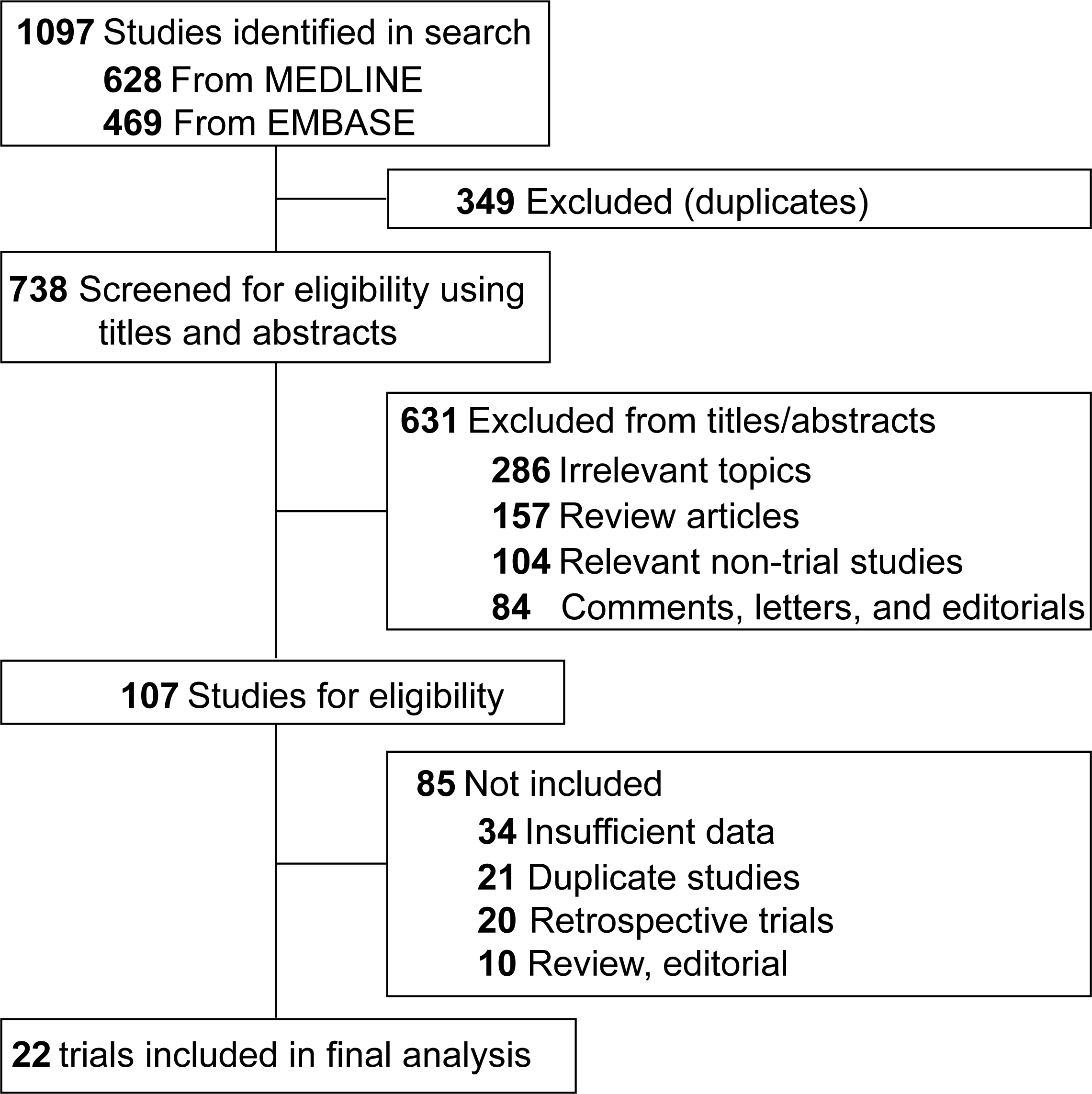

Supplement: Supplementary Figure 1 — Flow-chart diagram of selected clinical trials included in our study. [file Image1.tif]

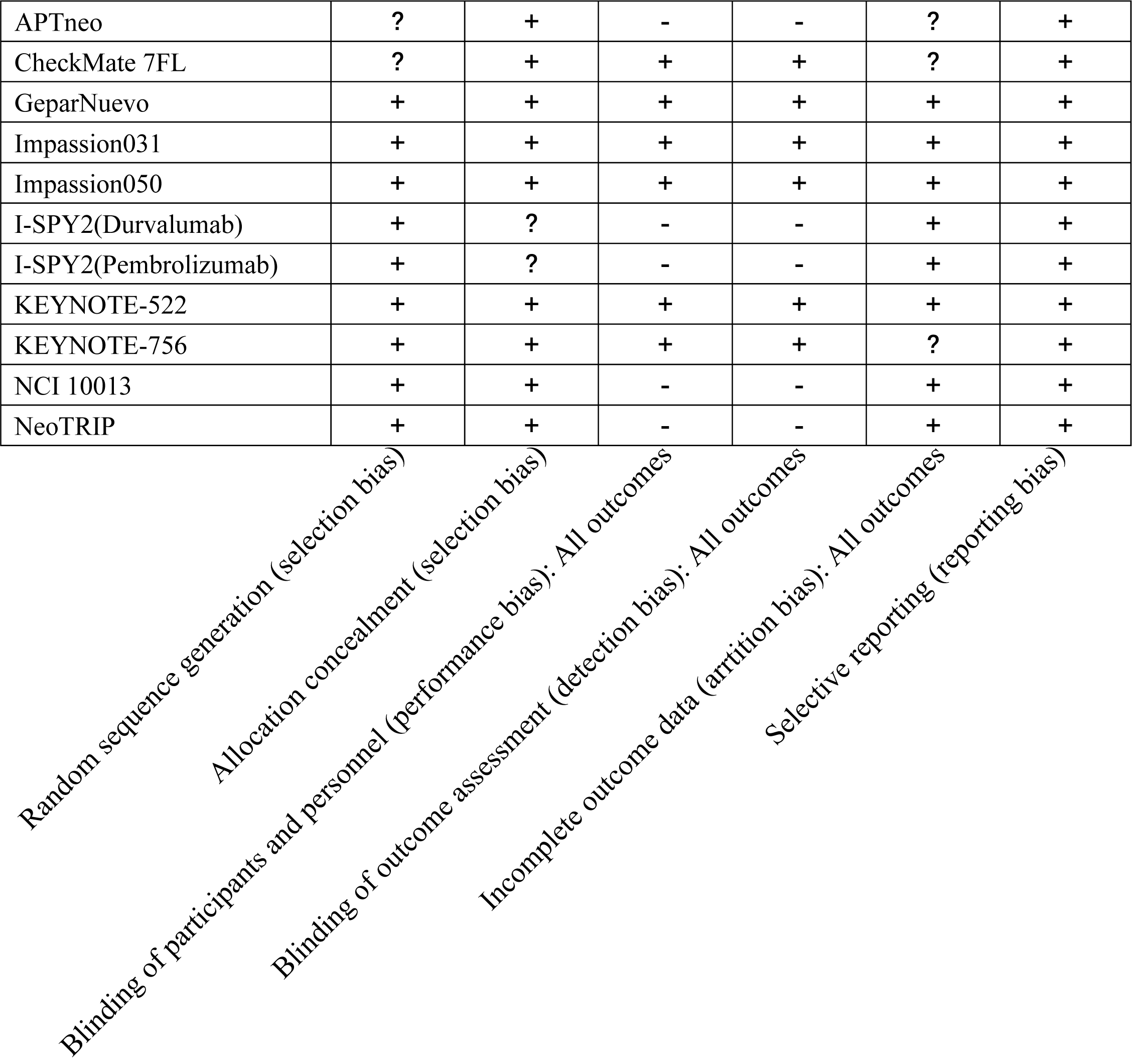

Supplement: Supplementary Figure 2 — Risk of bias of eligible randomized trials assessed by Cochrane risk of bias tool. [file Image2.tif]

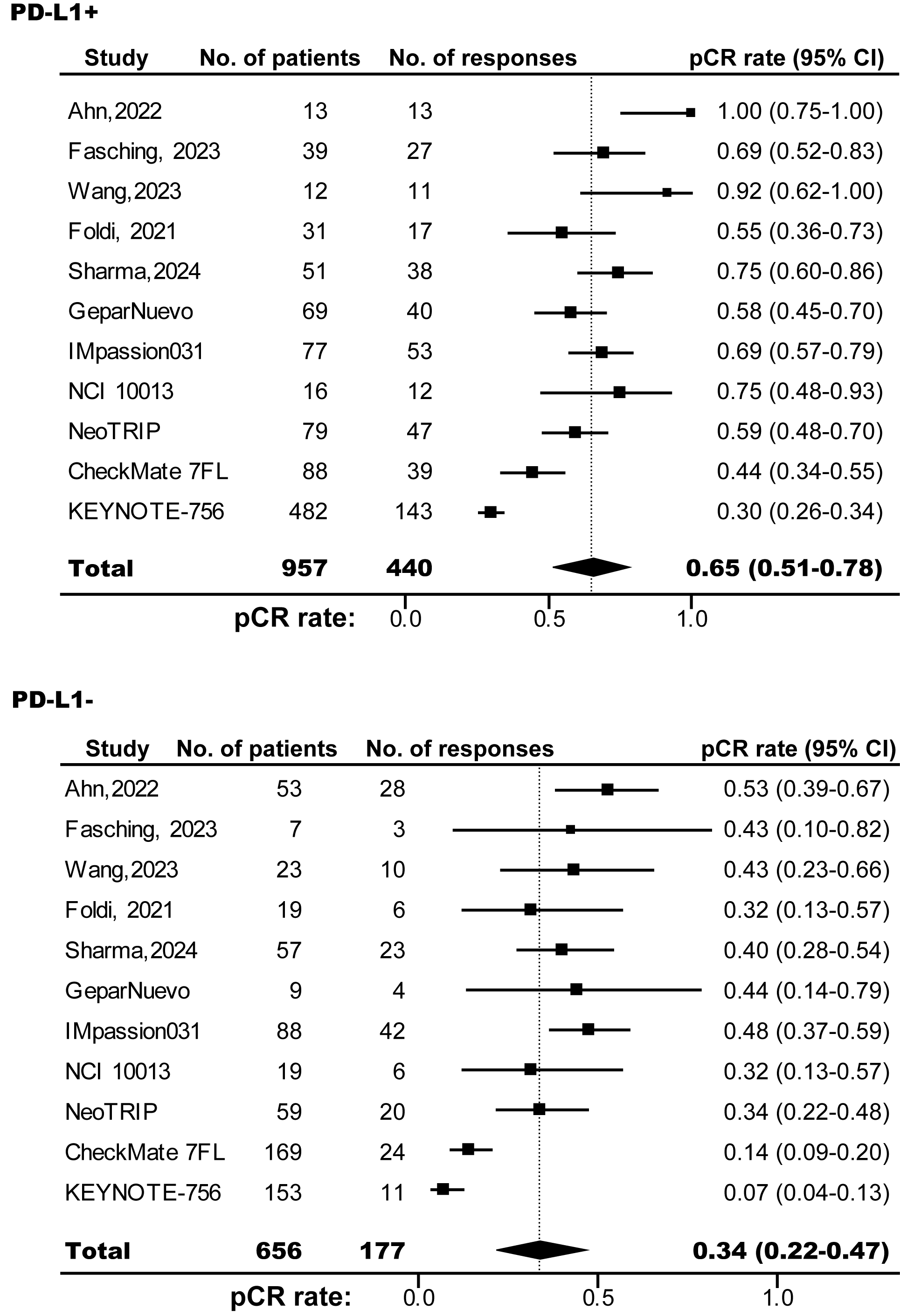

Supplement: Supplementary Figure 3 — The pooled pCR rates in patients with PD-L1 positive BC and patients with PD-L1 negative BC. The vertical dot line indicates the overall pCR rate in patients with PD-L1 positive BC and patients with PD-L1 negative BC. BC, breast cancer; pCR, pathological complete response. [file Image3.tif]

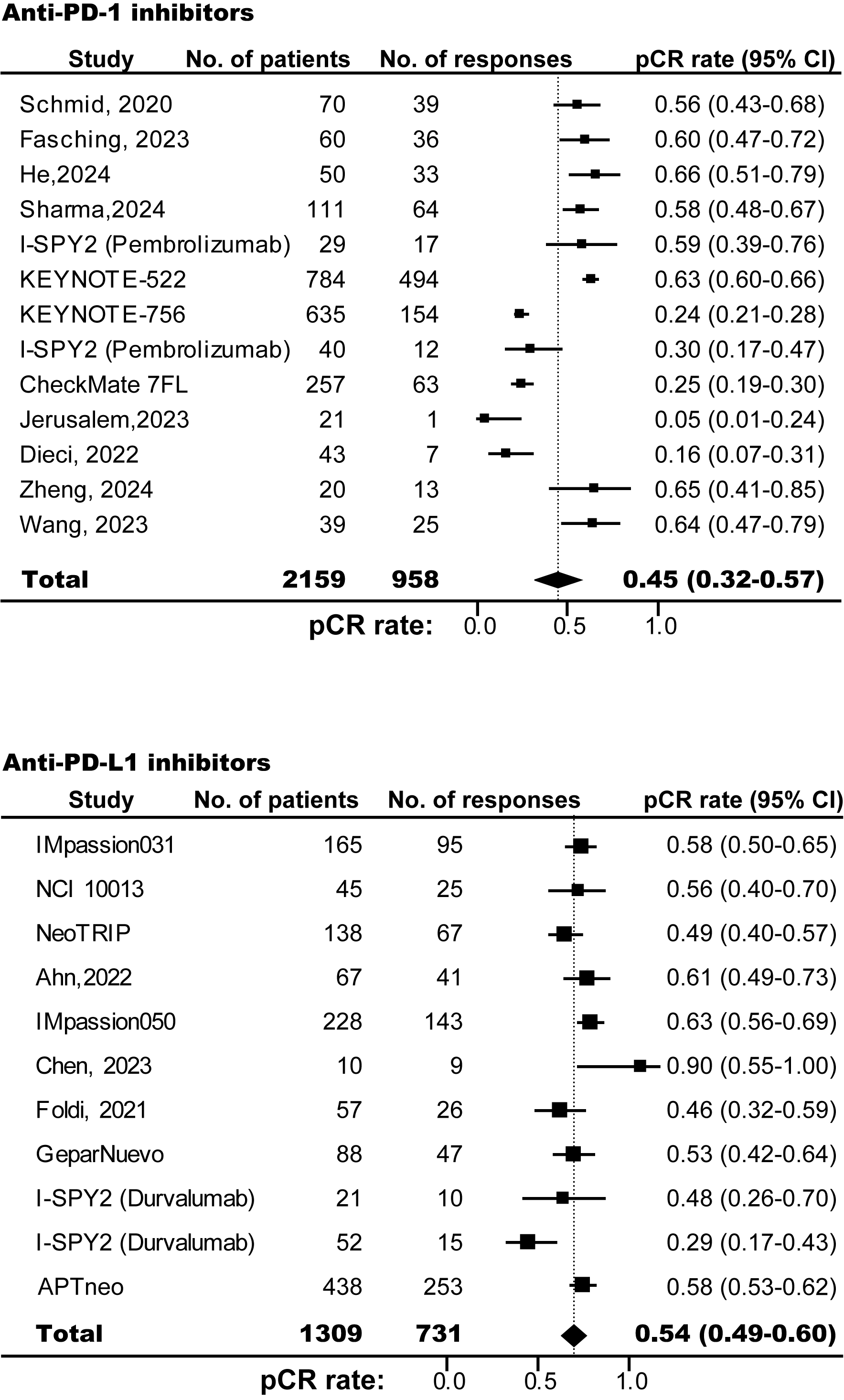

Supplement: Supplementary Figure 4 — The pooled pCR rates in BC patients treated with anti-PD-L1-based and anti-PD-1-based neoadjuvant settings. The vertical dot line indicates the overall pCR rate in patients treated with anti-PD-L1-based and anti-PD-1-based neoadjuvant settings. BC, breast cancer; pCR, pathological complete response. [file Image4.tif]

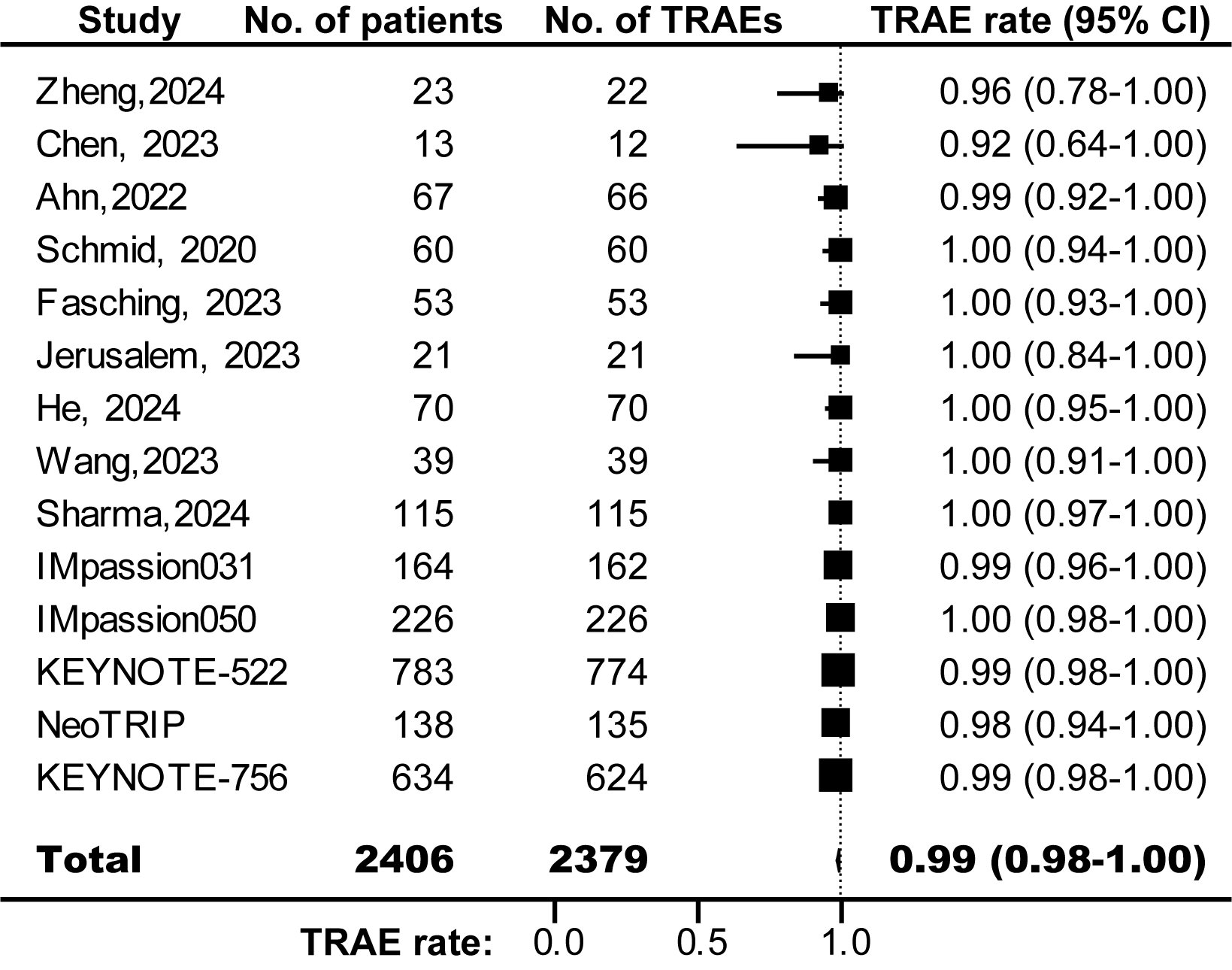

Supplement: Supplementary Figure 5 — The pooled any-grade TRAE rate in BC patients treated with ICB-based neoadjuvant regimens. The vertical dot line indicates the overall any-grade TRAE rate. BC, breast cancer; ICB, immune checkpoint inhibitor; TRAE, treatment-related adverse event. [file Image5.tif]

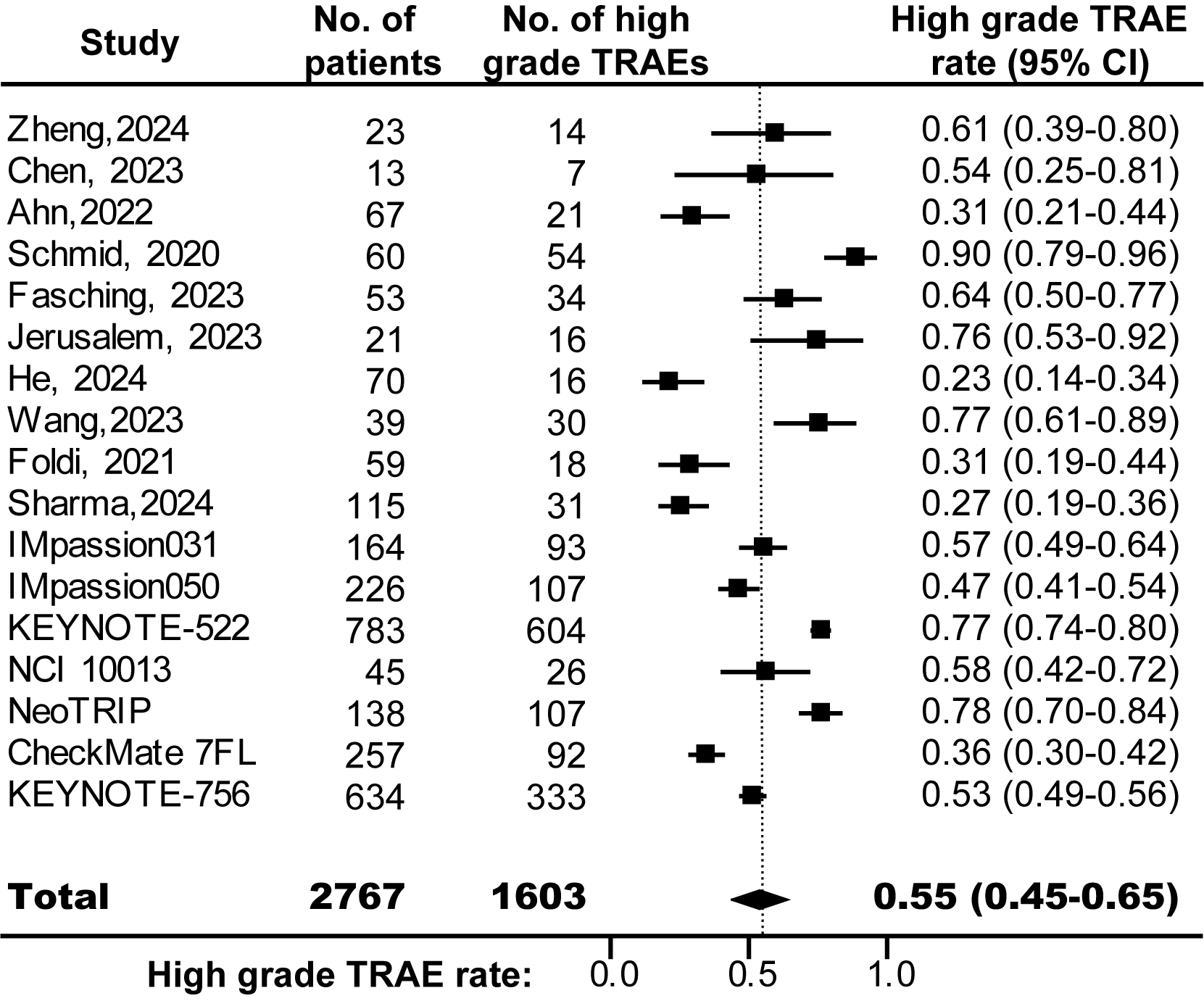

Supplement: Supplementary Figure 6 — The pooled high-grade TRAE rate in BC patients treated with ICB-based neoadjuvant regimens. The vertical dot line indicates the overall high-grade TRAE rate. BC, breast cancer; ICB, immune checkpoint inhibitor; TRAE, treatment-related adverse event. [file Image6.tif]

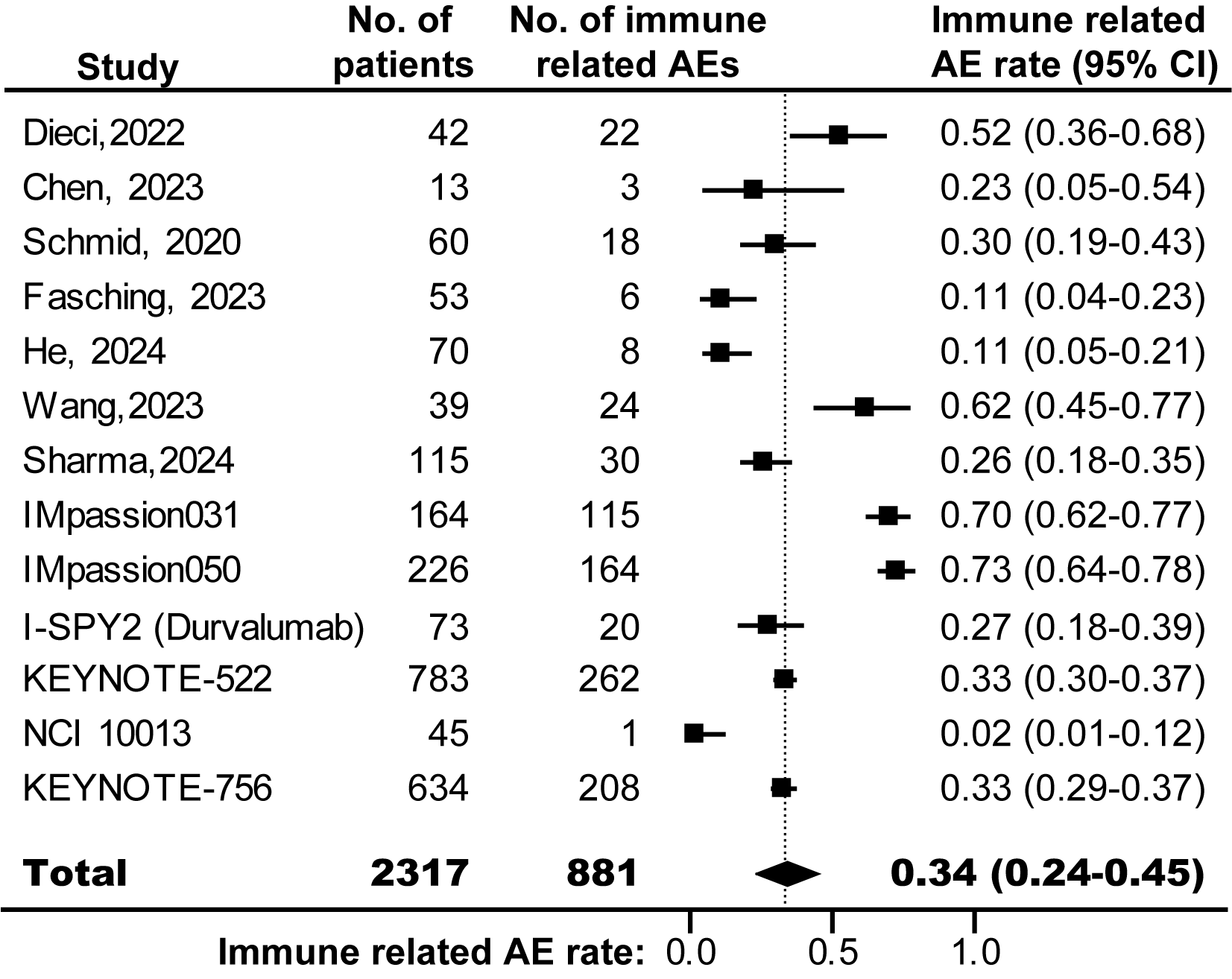

Supplement: Supplementary Figure 7 — The pooled any-grade irAE rate in BC patients treated with ICB-based neoadjuvant regimens. The vertical dot line indicates the overall any-grade irAE rate. BC, breast cancer; ICB, immune checkpoint inhibitor; irAE, immune-related adverse event. [file Image7.tif]

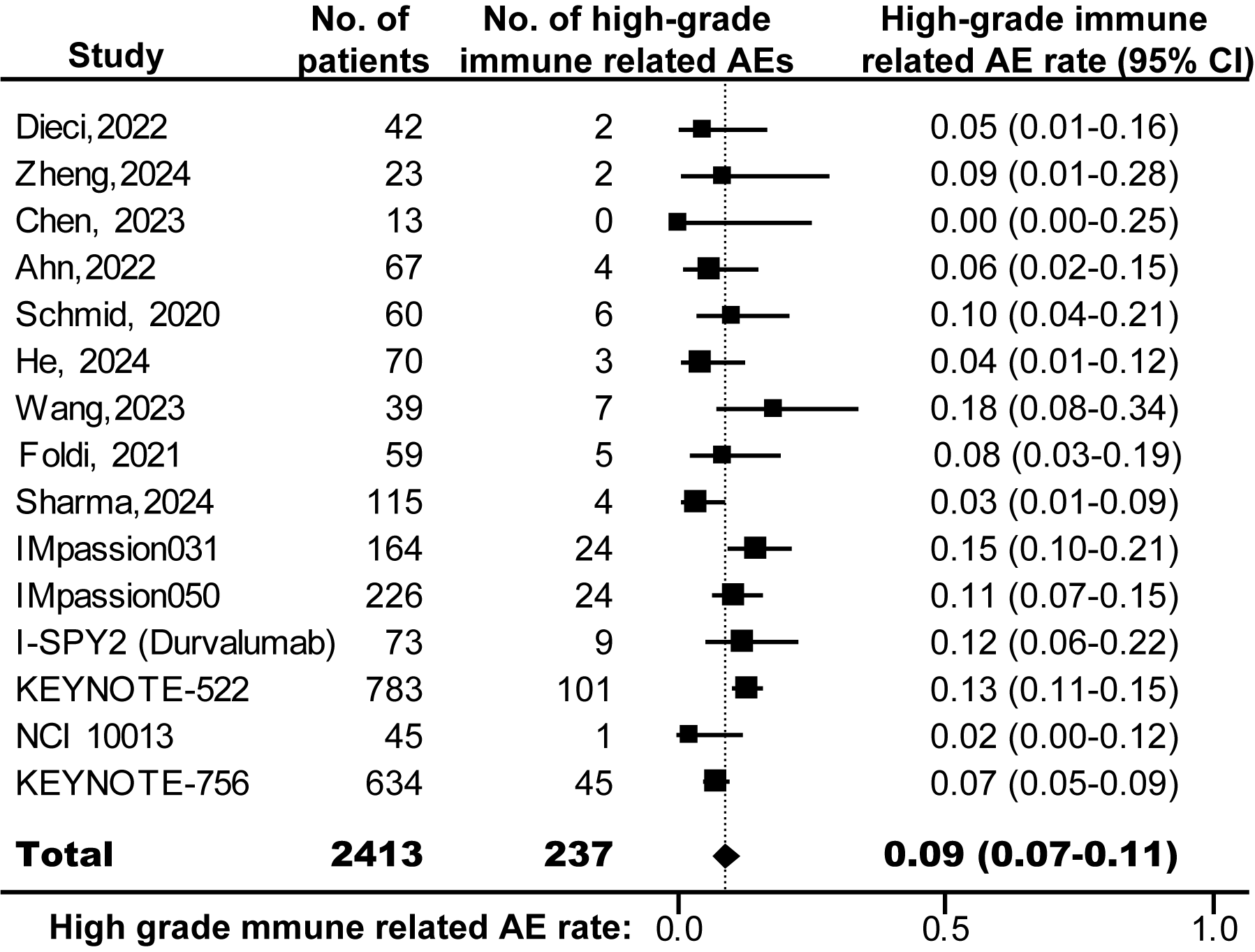

Supplement: Supplementary Figure 8 — The pooled high-grade irAE rate in BC patients treated with ICB-based neoadjuvant regimens. The vertical dot line indicates the overall any-grade irAE rate. BC, breast cancer; ICB, immune checkpoint inhibitor; irAE, immune-related adverse event. [file Image8.tif]

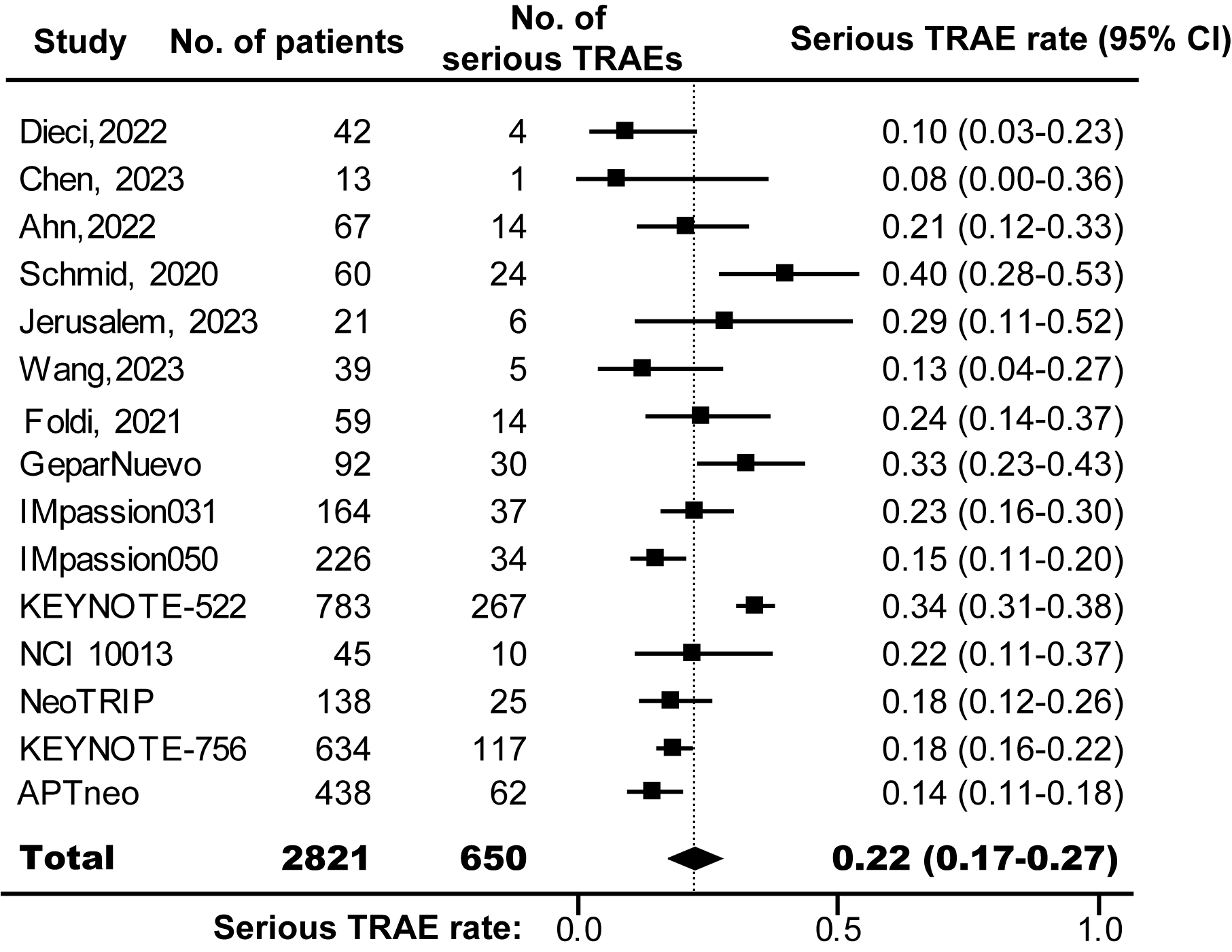

Supplement: Supplementary Figure 9 — The pooled serious TRAE rate in BC patients treated with ICB-based neoadjuvant regimens. The vertical dot line indicates the overall high-grade TRAE rate. BC, breast cancer; ICB, immune checkpoint inhibitor; TRAE, treatment-related adverse event. [file Image9.tif]

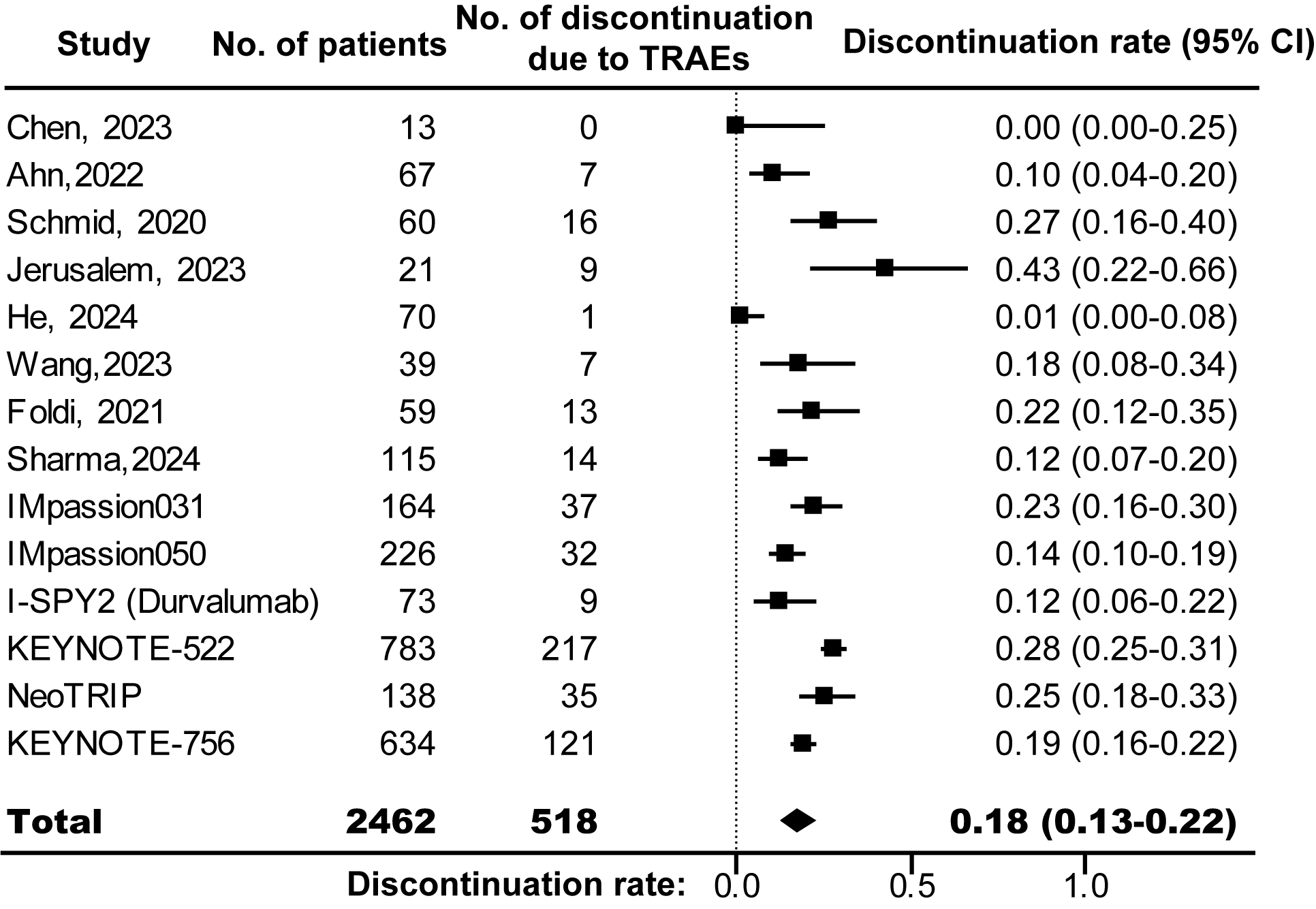

Supplement: Supplementary Figure 10 — The pooled incidence of treatment discontinuation due to TRAE in BC patients treated with ICB-based neoadjuvant regimens. The vertical dot line indicates the overall treatment discontinuation rate. BC, breast cancer; ICB, immune checkpoint inhibitor; TRAE, treatment-related adverse event. [file Image10.tif]

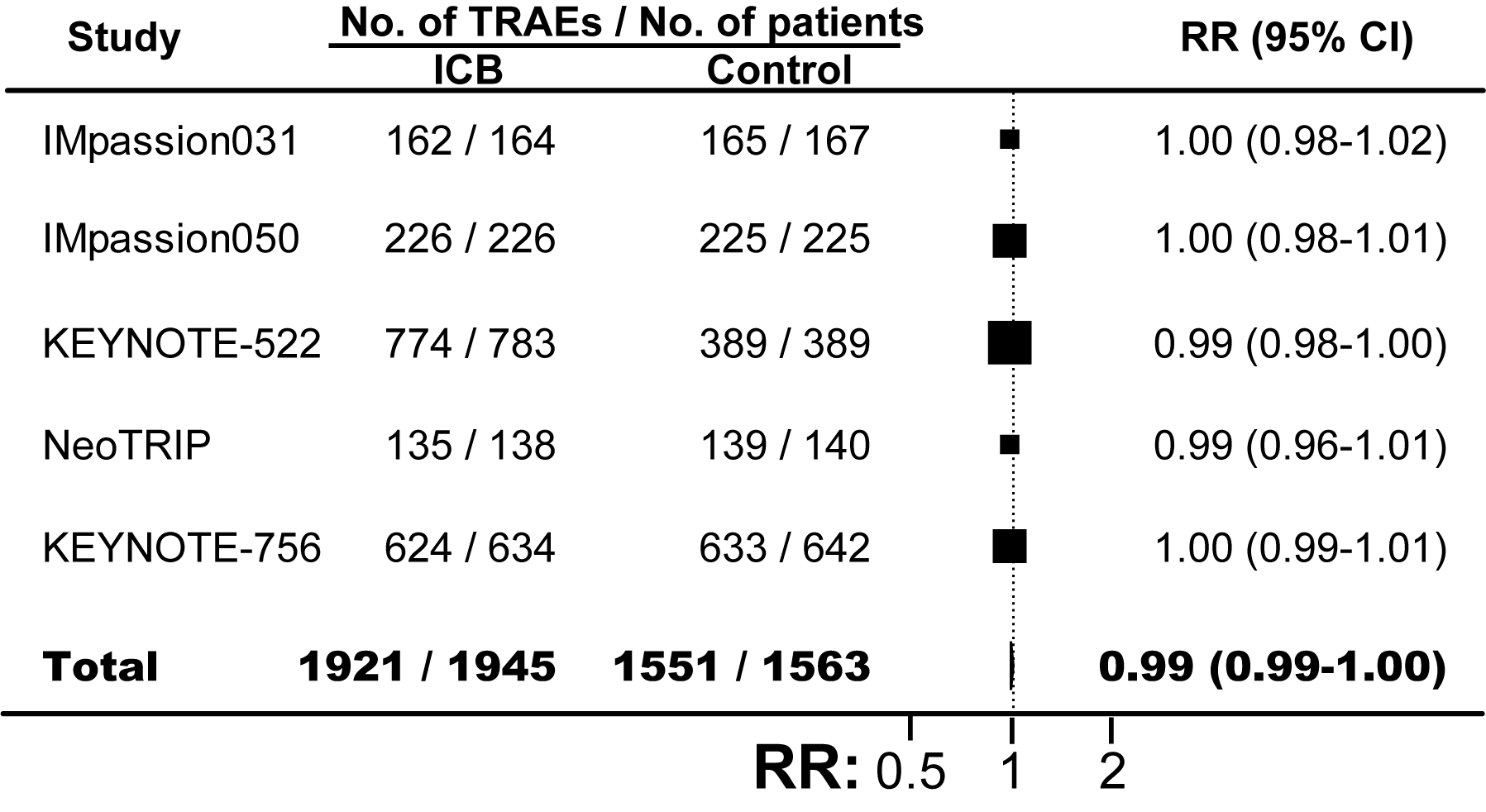

Supplement: Supplementary Figure 11 — The pooled relative risk of any-grade TRAE in BC patients treated with ICB-based neoadjuvant regimens. The vertical dot line indicates 1. BC, breast cancer; ICB, immune checkpoint inhibitor; RR, relative risk; TRAE, treatment-related adverse event. [file Image11.tif]

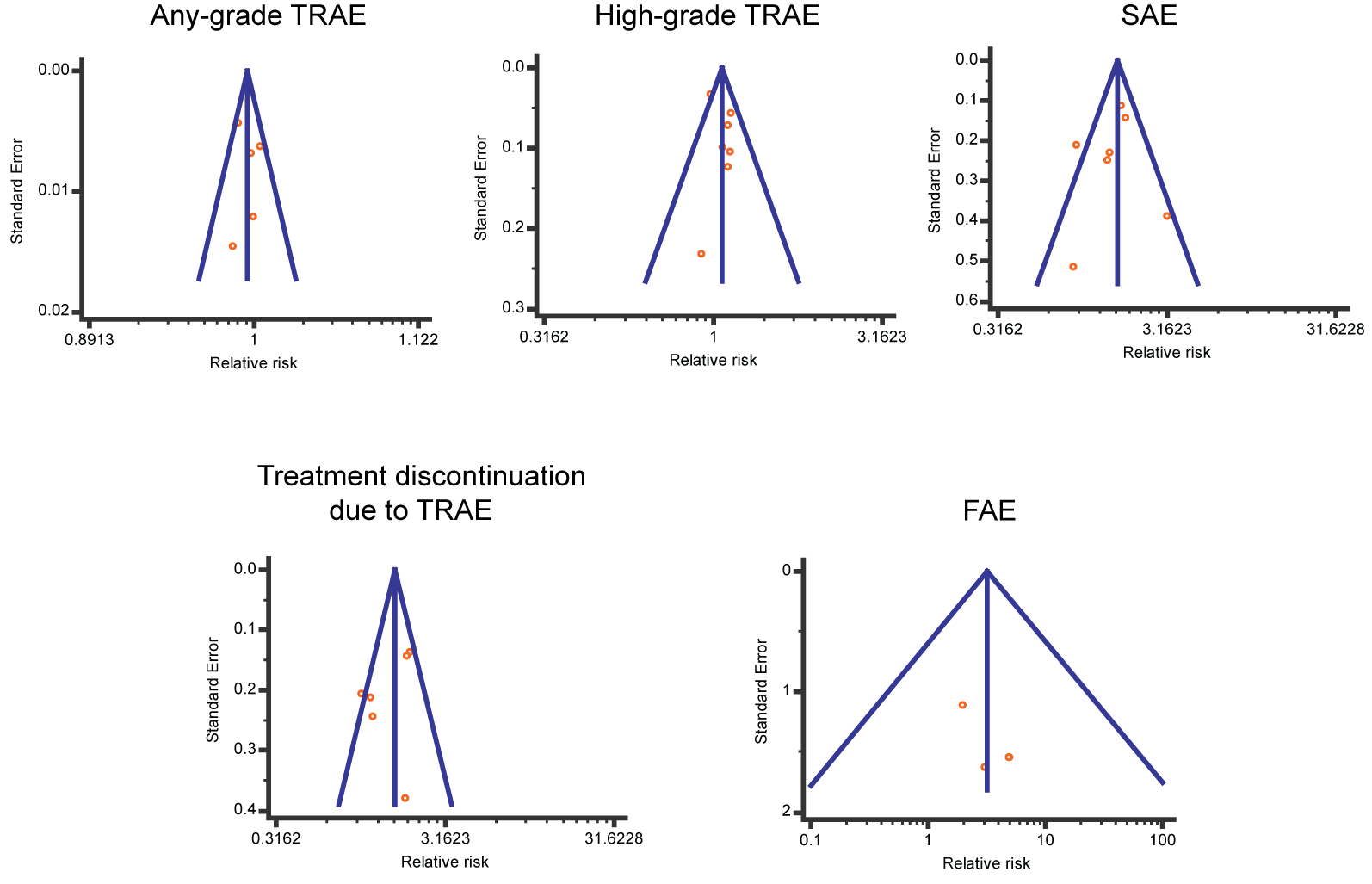

Supplement: Supplementary Figure 12 — Begg’s funnel plot for the publication bias test. Each circle represents a separate trial for the indicated association. Vertical line, mean effect size. [file Image12.tif]
