# Supplementary material for: A system review of neoadjuvant immune checkpoint blockade for breast cancer
Source: Front Immunol. 2025 Mar 27;16:1537926. doi: 10.3389/fimmu.2025.1537926 (PMC11983617; doi:10.3389/fimmu.2025.1537926)
Supplement: Supplementary file 13 [file Table1.docx]

**Table S1. Baseline characteristics of eligible non-randomized trials.**

| **Study** | **Registered identification** | **Phase** | **cancer type** | **Treatment** | **No. of patients** | **Median age (range, year)** | **No. of PD-L1+/PD-L1-** | **Primary endpoint** |
| --- | --- | --- | --- | --- | --- | --- | --- | --- |
| Dieci,2022^1^ | NCT04659551 | II | HR+/HER2- | Nivolumab + epirubicin/cyclophosphamide | 43 | 45(31-54) | NA | pCR |
| Zheng,2024^2^ | NCT04676997 | II | TNBC | Camrelizumab + nonplatinum-based chemotherapy | 23 | 52 (29–65) | NA | pCR |
| Chen, 2023^3^ | NCT05132790 | NA | TNBC | Adebrelimab + radiotherapy + chemotherapy | 13 | 51 (31–68) | NA | pCR |
| Ahn,2022^4^ | NCT03881878 | II | HER2+ | Atezolizumab + pertuzumab + docetaxel + trastuzumab | 67 | 52 (33-74) | 13/53 | pCR |
| Schmid, 2020^5^ | NCT02622074 | I | TNBC | Pembrolizumab +chemotherapy | 60 | 49 (26-71) | NA | Safety |
| Fasching, 2023^6^ | NCT03289819 | II | TNBC | Pembrolizumab + nab-paclitaxel | 50 | 50 | 39/7 | pCR |
| Jerusalem, 2023^7^ | NCT04075604 | I/II | ER+/HER2- | Nivolumab + palbociclib + anastrozole | 21 | 65(45-80) | NA | Safety |
| He, 2024^8^ | NCT04418154 | II | TNBC | Toripalimab + nab-paclitaxel | 70 | 51 (23–70) | NA | pCR |
| Wang,2023^9^ | NCT04213898 | II | TNBC | Camrelizumab + nab-paclitaxel + epirubicin | 39 | 46(31-59) | 12/23 | pCR |
| Foldi, 2021^10^ | NCT02489448 | I/II | TNBC | Durvalumab+ nab-paclitaxel + doxorubicin/cyclophosphamide | 59 | 50 | 31/19 | pCR |
| Sharma,2024^11^ | NCT03639948 | II | TNBC | Pembrolizumab + anthracycline-based chemotherapy | 115 | 50(27-70) | 51/57 | pCR |

**Table S2. The definition of PD-L1 positivity in eligible trials.**

| **Study** | **Study design** | **Antibody Clone** | **Scoring standard** | **Cut-off value** |
| --- | --- | --- | --- | --- |
| Ahn,2022^4^ | Non-randomized | SP142 Ventana | IC score | IC ≥1% |
| APTneo^12^ | Randomized | SP142 Ventana | IC score | IC ≥1% |
| Impassion031^13,14^ | Randomized | SP142 Ventana | IC score | IC ≥1% |
| Impassion050^15,16^ | Randomized | SP142 Ventana | IC score | IC ≥1% |
| NCI 10013^17^ | Randomized | SP142 Ventana | IC score | IC ≥1% |
| NeoTRIP^18,19^ | Randomized | SP142 Ventana | IC score | IC ≥1% |
| Fasching, 2023^6^ | Non-randomized | 22C3 pharmDx Dako | CPS score | CPS ≥ 10 |
| Sharma,2024^11^ | Non-randomized | 22C3 pharmDx Dako | CPS score | CPS ≥ 10 |
| Keynote-522^20-22^ | Randomized | 22C3 pharmDx Dako | CPS score | CPS ≥ 1 |
| KEYNOTE-756^23^ | Randomized | 22C3 pharmDx Dako | CPS score | CPS ≥ 1 |
| Wang,2023^9^ | Non-randomized | 22C3 pharmDx Dako | CPS score | CPS ≥ 1 |
| Foldi, 2021^10^ | Non-randomized | SP263 Ventana | TC or IC score | TC or IC score ≥1% |
| GeparNuevo^24,25^ | Randomized | SP263 Ventana | TC or IC score | TC or IC score ≥1% |
| CheckMate 7FL^26^ | Randomized | 28-8 pharmDx Dako | CPS score | CPS ≥ 1 |

CPS: combined positive score; IC: immune cell; TC: tumor cell

**Table S3. Johanna Brigs bias evaluation for 11 eligible non-randomized trials.**

| **Study** | **1. Were the two groups similar and recruited from the same population?** | **2. Were the exposures measured similarly to both exposed and unexposed groups?** | **3. Was the exposure measured in a valid and reliable way?** | **4. Were confounding factors identified?** | **5. Were strategies to deal with confounding factors stated?** | **6. Were the groups/participants free of the outcome at the start of the study?** | **7. Were the outcomes measured in a valid and reliable way?** | **8. Was the follow up time reported and sufficient to be long enough for outcomes to occur?** | **9. Was follow up complete, and if not, were the reasons described and explored?** | **10. Were strategies to address incomplete follow up utilized?** | **11. Was appropriate statistical analysis used?** |
| --- | --- | --- | --- | --- | --- | --- | --- | --- | --- | --- | --- |
| Dieci,2022^1^ | N/A | N/A | Y | N | N | Y | Y | N | N | U | Y |
| Zheng,2024^2^ | N/A | N/A | Y | N | N | Y | Y | N | N | U | Y |
| Chen, 2023^3^ | N/A | N/A | Y | N | N | Y | Y | U | N | N | Y |
| Ahn,2022^4^ | N/A | N/A | Y | Y | U | Y | Y | U | U | U | Y |
| Schmid, 2020^5^ | N/A | N/A | Y | Y | Y | Y | Y | Y | Y | N | Y |
| Fasching, 2023^6^ | N/A | N/A | Y | N | N | Y | Y | N | N | N | Y |
| Jerusalem, 2023^7^ | N/A | N/A | Y | Y | N | Y | Y | U | N | N | Y |
| He, 2024^8^ | N/A | N/A | Y | Y | Y | Y | Y | Y | Y | U | Y |
| Wang,2023^9^ | N/A | N/A | Y | N | N | Y | Y | N | N | N | Y |
| Foldi, 2021^10^ | N/A | N/A | Y | Y | U | Y | Y | N | N | N | Y |
| Sharma,2024^11^ | N/A | N/A | Y | N | N | Y | Y | Y | Y | U | Y |

**References:**

1. Dieci MV, Guarneri V, Tosi A, et al. Neoadjuvant Chemotherapy and Immunotherapy in Luminal B-like Breast Cancer: Results of the Phase II GIADA Trial. *Clinical cancer research : an official journal of the American Association for Cancer Research.* 2022;28(2):308-317.

2. Zheng C, Liu Y, Wang X, et al. Clinical efficacy and biomarker analysis of neoadjuvant camrelizumab plus chemotherapy for early-stage triple-negative breast cancer: a experimental single-arm phase II clinical trial pilot study. *International journal of surgery (London, England).* 2024;110(3):1527-1536.

3. Chen G, Gu X, Xue J, et al. Effects of neoadjuvant stereotactic body radiotherapy plus adebrelimab and chemotherapy for triple-negative breast cancer: A pilot study. *eLife.* 2023;12.

4. Ahn HK, Sim SH, Suh KJ, et al. Response Rate and Safety of a Neoadjuvant Pertuzumab, Atezolizumab, Docetaxel, and Trastuzumab Regimen for Patients With ERBB2-Positive Stage II/III Breast Cancer: The Neo-PATH Phase 2 Nonrandomized Clinical Trial. *JAMA oncology.* 2022;8(9):1271-1277.

5. Schmid P, Salgado R, Park YH, et al. Pembrolizumab plus chemotherapy as neoadjuvant treatment of high-risk, early-stage triple-negative breast cancer: results from the phase 1b open-label, multicohort KEYNOTE-173 study. *Annals of oncology : official journal of the European Society for Medical Oncology.* 2020;31(5):569-581.

6. Fasching PA, Hein A, Kolberg HC, et al. Pembrolizumab in combination with nab-paclitaxel for the treatment of patients with early-stage triple-negative breast cancer - A single-arm phase II trial (NeoImmunoboost, AGO-B-041). *European journal of cancer (Oxford, England : 1990).* 2023;184:1-9.

7. Jerusalem G, Prat A, Salgado R, et al. Neoadjuvant nivolumab + palbociclib + anastrozole for oestrogen receptor-positive/human epidermal growth factor receptor 2-negative primary breast cancer: Results from CheckMate 7A8. *Breast (Edinburgh, Scotland).* 2023;72:103580.

8. He M, Hao S, Ma L, et al. Neoadjuvant anthracycline followed by toripalimab combined with nab-paclitaxel in patients with early triple-negative breast cancer (NeoTENNIS): a single-arm, phase II study. *EClinicalMedicine.* 2024;74:102700.

9. Wang C, Liu Z, Chen X, et al. Neoadjuvant camrelizumab plus nab-paclitaxel and epirubicin in early triple-negative breast cancer: a single-arm phase II trial. *Nature communications.* 2023;14(1):6654.

10. Foldi J, Silber A, Reisenbichler E, et al. Neoadjuvant durvalumab plus weekly nab-paclitaxel and dose-dense doxorubicin/cyclophosphamide in triple-negative breast cancer. *NPJ breast cancer.* 2021;7(1):9.

11. Sharma P, Stecklein SR, Yoder R, et al. Clinical and Biomarker Findings of Neoadjuvant Pembrolizumab and Carboplatin Plus Docetaxel in Triple-Negative Breast Cancer: NeoPACT Phase 2 Clinical Trial. *JAMA oncology.* 2024;10(2):227-235.

12. Gianni L, Munzone E, Mansutti M, et al. Abstract LBO1-02: Pathologic complete response (pCR) of neoadjuvant therapy with or without atezolizumab in HER2-positive, early high-risk and locally advanced breast cancer: APTneo Michelangelo randomized trial. *Cancer Research.* 2024;84(9_Supplement):LBO1-02-LBO01-02.

13. Mittendorf EA, Zhang H, Barrios CH, et al. Neoadjuvant atezolizumab in combination with sequential nab-paclitaxel and anthracycline-based chemotherapy versus placebo and chemotherapy in patients with early-stage triple-negative breast cancer (IMpassion031): a randomised, double-blind, phase 3 trial. *Lancet (London, England).* 2020;396(10257):1090-1100.

14. Barrios C, Harbeck N, Zhang HA, et al. LBA1 Final analysis of the placebo-controlled randomised phase III IMpassion031 trial evaluating neoadjuvant atezolizumab (atezo) plus chemotherapy (CT) followed by open-label adjuvant atezo in patients (pts) with early-stage triple-negative breast cancer (eTNBC). *ESMO Open.* 2023;8(1).

15. Huober J, Barrios CH, Niikura N, et al. Atezolizumab With Neoadjuvant Anti-Human Epidermal Growth Factor Receptor 2 Therapy and Chemotherapy in Human Epidermal Growth Factor Receptor 2-Positive Early Breast Cancer: Primary Results of the Randomized Phase III IMpassion050 Trial. *Journal of clinical oncology : official journal of the American Society of Clinical Oncology.* 2022;40(25):2946-2956.

16. Huober J, Barrios CH, Niikura N, et al. 127P Atezolizumab (A) + pertuzumab + trastuzumab (PH) + chemotherapy (CT) in HER2-positive early breast cancer (HER2+ eBC): Final results of the phase III IMpassion050 trial. *ESMO Open.* 2024;9.

17. Ademuyiwa FO, Gao F, Street CR, et al. A randomized phase 2 study of neoadjuvant carboplatin and paclitaxel with or without atezolizumab in triple negative breast cancer (TNBC) - NCI 10013. *NPJ breast cancer.* 2022;8(1):134.

18. Gianni L, Huang CS, Egle D, et al. Pathologic complete response (pCR) to neoadjuvant treatment with or without atezolizumab in triple-negative, early high-risk and locally advanced breast cancer: NeoTRIP Michelangelo randomized study. *Annals of oncology : official journal of the European Society for Medical Oncology.* 2022;33(5):534-543.

19. Gianni L, Huang C, Egle D, et al. LBA19 Event-free survival (EFS) analysis of neoadjuvant taxane/carboplatin with or without atezolizumab followed by an adjuvant anthracycline regimen in high-risk triple negative breast cancer (TNBC): NeoTRIP Michelangelo randomized study. *Annals of Oncology.* 2023;34:S1258-S1259.

20. Schmid P, Cortes J, Dent R, et al. Event-free Survival with Pembrolizumab in Early Triple-Negative Breast Cancer. *The New England journal of medicine.* 2022;386(6):556-567.

21. Shah M, Osgood CL, Amatya AK, et al. FDA Approval Summary: Pembrolizumab for Neoadjuvant and Adjuvant Treatment of Patients with High-Risk Early-Stage Triple-Negative Breast Cancer. *Clinical cancer research : an official journal of the American Association for Cancer Research.* 2022;28(24):5249-5253.

22. Pusztai L, Denkert C, O'Shaughnessy J, et al. Event-free survival by residual cancer burden with pembrolizumab in early-stage TNBC: exploratory analysis from KEYNOTE-522. *Annals of oncology : official journal of the European Society for Medical Oncology.* 2024;35(5):429-436.

23. Cardoso F, McArthur HL, Schmid P, et al. LBA21 KEYNOTE-756: Phase III study of neoadjuvant pembrolizumab (pembro) or placebo (pbo) + chemotherapy (chemo), followed by adjuvant pembro or pbo + endocrine therapy (ET) for early-stage high-risk ER+/HER2&#x2013; breast cancer. *Annals of Oncology.* 2023;34:S1260-S1261.

24. Loibl S, Schneeweiss A, Huober J, et al. Neoadjuvant durvalumab improves survival in early triple-negative breast cancer independent of pathological complete response. *Annals of oncology : official journal of the European Society for Medical Oncology.* 2022;33(11):1149-1158.

25. Loibl S, Untch M, Burchardi N, et al. A randomised phase II study investigating durvalumab in addition to an anthracycline taxane-based neoadjuvant therapy in early triple-negative breast cancer: clinical results and biomarker analysis of GeparNuevo study. *Annals of oncology : official journal of the European Society for Medical Oncology.* 2019;30(8):1279-1288.

26. Loi S, Curigliano G, Salgado RF, et al. LBA20 A randomized, double-blind trial of nivolumab (NIVO) vs placebo (PBO) with neoadjuvant chemotherapy (NACT) followed by adjuvant endocrine therapy (ET) &#xb1; NIVO in patients (pts) with high-risk, ER+ HER2&#x2212; primary breast cancer (BC). *Annals of Oncology.* 2023;34:S1259-S1260.
